# Human bias and CNNs’ superior insights in satellite based poverty mapping

**DOI:** 10.1038/s41598-024-74150-9

**Published:** 2024-10-02

**Authors:** Hamid Sarmadi, Ibrahim Wahab, Ola Hall, Thorsteinn Rögnvaldsson, Mattias Ohlsson

**Affiliations:** 1 Department of Human Geography, Lund University, Lund, Sweden; 2 Centre for Applied Intelligent Systems Research (CAISR), Halmstad University, Halmstad, Sweden; 3 Centre for Environmental and Climate Science, Lund University, Lund, Sweden

**Keywords:** Welfare estimation, Satellite imagery, Domain experts, Human bias, Explainable AI, Convolutional neural networks, Tanzania, Computer science, Environmental economics, Socioeconomic scenarios

## Abstract

Satellite imagery is a potent tool for estimating human wealth and poverty, especially in regions lacking reliable data. This study compares a range of poverty estimation approaches from satellite images, spanning from expert-based to fully machine learning-based methodologies. Human experts ranked clusters from the Tanzania DHS survey using high-resolution satellite images. Then expert-defined features were utilized in a machine learning algorithm to estimate poverty. An explainability method was applied to assess the importance and interaction of these features in poverty prediction. Additionally, a convolutional neural network (CNN) was employed to estimate poverty from medium-resolution satellite images of the same locations. Our analysis indicates that increased human involvement in poverty estimation diminishes accuracy compared to machine learning involvement, exemplified with the case of Tanzania. Expert defined features exhibited significant overlap and poor interaction when used together in a classifier. Conversely, the CNN-based approach outperformed human experts, demonstrating superior predictive capability with medium-resolution images. These findings highlight the importance of leveraging machine learning explainability methods to identify predictive elements that may be overlooked by human experts. This study advocates for the integration of emerging technologies with traditional methodologies to optimize data collection and analysis of poverty and welfare.

**Supplementary Information:**

The online version contains supplementary material available at 10.1038/s41598-024-74150-9.

## Introduction

The quest to reduce and eventually eradicate poverty stands as a pillar of global development, outlined in the United Nations’ Sustainable Development Goals^1^. Central to this mission is the ambition that no individual or community should be left in destitution. Poverty is fundamentally about a lack of basic necessities and opportunities to improve one’s life condition, often quantified by income levels, access to education, and healthcare. In contrast, welfare refers to the well-being of individuals and groups, encompassing not only material and economic conditions but also broader aspects like health, education, and employment security. A critical step towards the eradication of poverty lies in the precise measurement and identification of poverty-stricken regions, villages, and households, a process pivotal to prioritizing development assistance^2^. While developed regions frequently employ income-based methodologies to gauge welfare, due in part to the ready availability of robust income data, such strategies are less viable in many developing countries. There, income records are often incomplete or entirely absent, rendering them an unreliable indicator. Likewise, expenditure-based welfare assessments are compromised by the predominance of cash transactions in these economies and are further susceptible to seasonal fluctuations, detracting from their reliability in assessing poverty^3^.

In light of these challenges, the asset-based approach to poverty measurement has gained prominence, offering a more reliable measure of household and neighborhood welfare^2^. Among the key tools in this approach is the Demographic and Health Surveys (DHS) Asset Wealth Index, which constructs an indicator of a household’s long-term wealth based on its ownership of durable assets (like televisions and bicycles), housing characteristics (such as the presence of electricity and type of flooring), and access to services (e.g., source of drinking water). This index does not measure income or expenditure directly but infers wealth from the accumulation of assets, providing a more stable measure of economic status that is less susceptible to short-term fluctuations than income^4^.

The DHS Asset Wealth Index is particularly effective in capturing differences in wealth within populations, thereby aiding in the identification of poverty-stricken areas. By classifying households into wealth quintiles, ranging from poorest to richest, this index helps policymakers and researchers discern not only the depth and breadth of poverty but also to understand patterns of inequality and economic stratification. It thereby plays a crucial role in both the targeting of developmental assistance and in the monitoring of progress towards the reduction of poverty.

Against this backdrop of traditional methodologies, the field of artificial intelligence has made remarkable advances, particularly in developing algorithms that surpass human performance in complex tasks. Notable achievements include DeepMind’s AlphaGo’s victory over Go champion Lee Sedol and OpenAI’s algorithms achieving grandmaster status in Chess^5^. They have reached a level of sophistication that they are now used for learning and developing the game. An intriguing and similar development is the analysis of satellite imagery through the lens of machine learning. These images provide a frequent and widespread data source, yet they are inherently unstructured and not tailored for discerning human conditions, being designed primarily to capture the physical landscape. Nevertheless, recent efforts to employ machine learning in this context have yielded promising results in estimating various forms of human poverty^6^. Such achievements signal a shift from classic strategies to more nuanced and sophisticated models like Convolutional Neural Networks (CNNs)^7^, which are crafted to emulate human cognitive patterns in pattern recognition.

This paper presents a novel study that contrasts domain expertise with the capabilities of a CNN in estimating poverty levels from satellite images. Our results demonstrate that the CNN, using lower resolution images, not only outperforms human evaluators but also reveals significant insights into the nature of bias and human learning potential. Recognizing the areas where human judgment is superseded by AI opens doors to enhancing our understanding of these intelligent systems and harnessing their capabilities to augment human decision-making processes and also learning potentially new insights about social phenomena.

## Results

Our findings indicate that human evaluators struggle to accurately assess wealth from high-resolution ($\sim 0.6$ m/pixel) satellite imagery. A common tendency observed is “centralizing,” where human-generated estimates gravitate towards the average wealth level rather than representing the actual distribution extremes. When humans are instructed to identify features they consider indicative of wealth, classifiers built upon these features perform notably better than mere human observation. This indicates that humans can well detect features that are important for wealth estimation, but they fail to combine them properly. However, the human feature selection process introduces its own biases, and it remains uncertain whether all pertinent features are accounted for, especially those that are complex or subtle in nature. In comparison, the CNN emerges as the most accurate tool for wealth estimation. The CNN’s ability to process and analyze the raw data ($\sim 10$ m/pixel) without preconceived notions results in the least biased outcome. The increasing introduction of human factors into the analysis correlates with greater bias and diminished accuracy. The implications of this is that CNNs could potentially serve as a means to aid human researchers in reducing their inherent biases. By integrating the objective analytical power of machine learning models, we might enhance human evaluative precision and expand the scope of their expertise. Below we follow with the details of our results. Complementary results are also presented in the Supplementary Material.

### Satellite imagery and wealth data

**Fig. 1:**
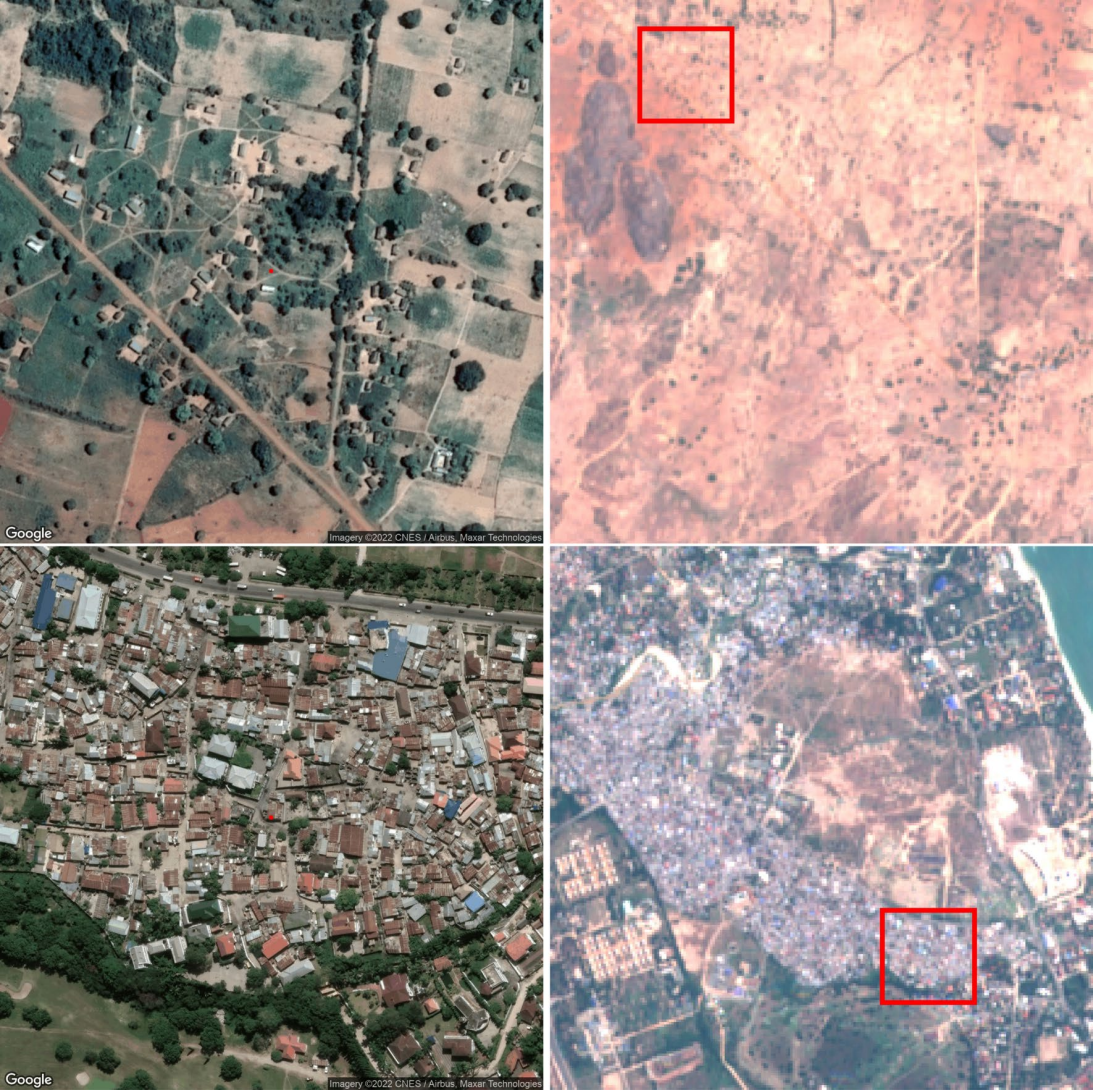
Examples of images shown to the domain experts (left) and input daylight images given to the CNN (right). The boundaries of the images to the left are shown as red squares in the images to the right. Images shown to domain experts were downloaded from Google Maps Static API (https://developers.google.com/maps/documentation/maps-static) and daylight images given to the CNN were downloaded using the Google Earth Engine API (https://developers.google.com/earth-engine) from the Sentinel-2 dataset, COPERNICUS/S2 image collection.

High-resolution images from Google API were used for the domain experts to view and classify into one of five wealth classes. A web page was setup for the experts where they were randomly assigned images to evaluate. The system allowed them to zoom in and out and move around in the image. The CNN was trained via transfer learning on 10 m/pixel-resolution images from Sentinel-2, with an area of about $2 \times 2$ square kilometers. Note that this is a lower resolution than has been used in earlier studies with CNNs, e.g.^8^. Figure 1 shows example images of the same locations, from Google Maps and from Sentinel-2. These images are combined with the relative wealth index from the sixth Demographic and Health Survey (DHS) data in Tanzania. This index is based on asset ownership, observations on housing quality, and access to services of households which have been grouped in quintiles ranging from ’poorest’ to ’richest’. We assigned the mean quintile of the housholds as representing the cluster. We then attempted to find the most likely location of each cluster, based on the DHS programme’s guidelines for anonymizing clusters and used these ’corrected’ location data to extract high-resolution satellite imagery for each cluster from the Google Maps Platform. Imagery for all 608 clusters was then loaded into our online survey portal for our domain experts to rate.

### Domain expert ratings

The 102 domain experts have significant experience (see Supplementary Table S.1). This is shown in not only their educational attainment and age distribution - 43% are 45 or more years old and 50% hold a Ph.D. - but even more important, half of the respondents ($N=51$) have more than 10 years of fieldwork experience in Sub-Saharan Africa (SSA). In terms of the region of experience, 48% have Tanzania, Eastern, or Southern Africa as their primary area of fieldwork experience.

**Fig. 2:**
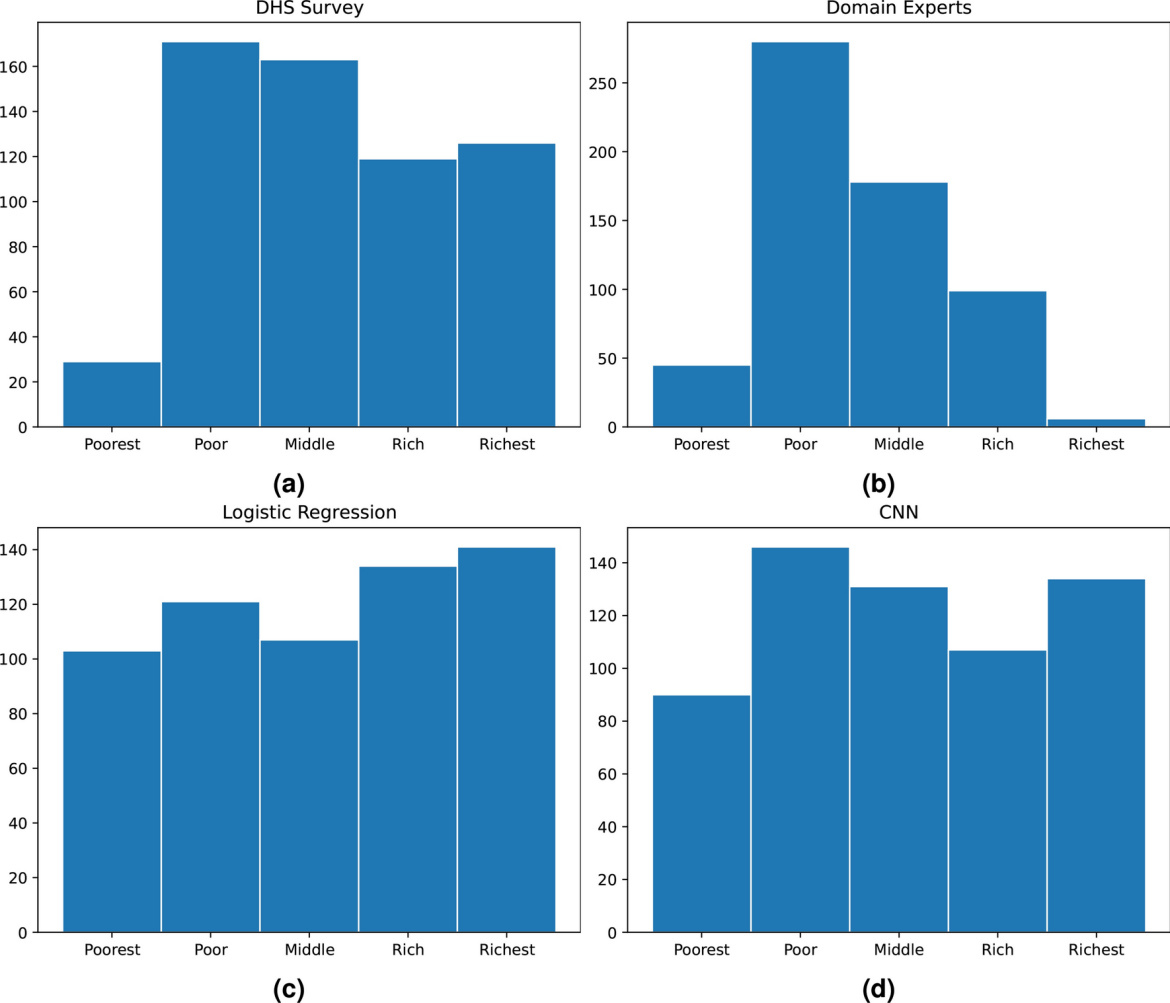
Wealth distribution at the cluster level from **a** the 2015 Tanzania DHS at the household level; **b** domain experts’ ratings; **c** logistic regression from expert-defined features; and **d** CNN based classifier, (N=608 clusters). CNN and logistic regression use balanced class weights for training to alleviate the sample imbalance across classes. The random forest, which is not shown, had problems with estimating the poorest class correctly.

**Fig. 3:**
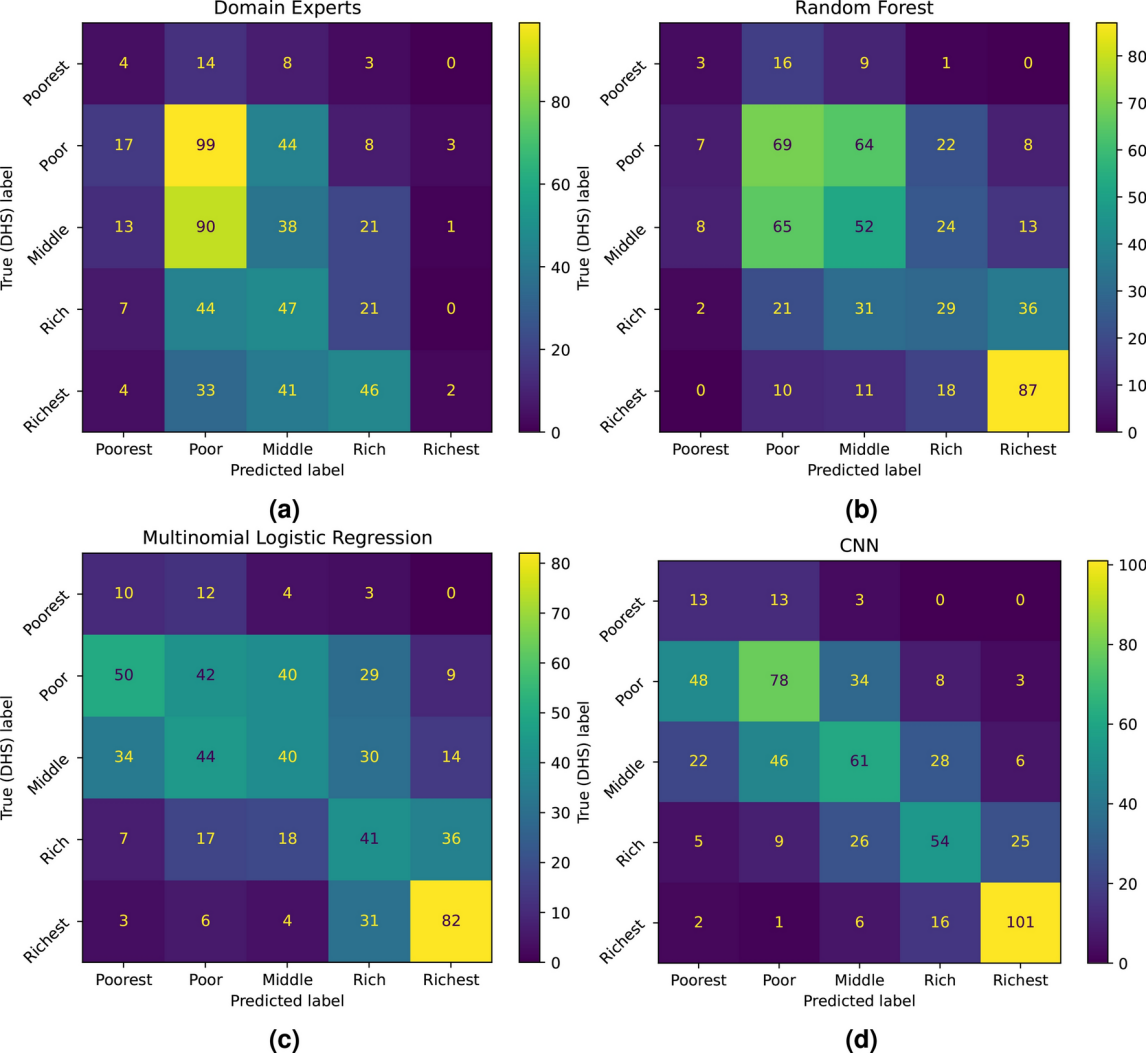
Confusion matrices for **a** domain experts, **b** random forest and **c** logistic regression using the expert-defined features, and **d** CNN using 10m/pixel satellite images. For random forest, logistic regression, and CNN; balanced class weights were used during training.

Figure 2a shows how the 608 sites’ wealth levels are distributed over the wealth quintile levels in the DHS data; this is the ground truth. Figure 2b shows the distribution of the wealth levels based on ratings by the domain experts. It is striking how the experts tend to prefer more average wealth levels, albeit biased towards low wealth. Furthermore, the domain experts are not only centralized in their estimates, they are also quite wrong. Figure 3a shows the confusion matrix between the domain experts’ estimates and the ground truth. The match is very poor, with a multiclass Matthews Correlation Coefficient (MCC) of 0.03 – 0.04, see Table 1, which is not significantly better than random guessing.

**Table 1:** Evaluation of domain experts, feature-based logistic regression, random forest, and CNN-based classifiers using Accuracy, Accuracy$\pm 1$, and MCC scores.

	Accuracy	Accuracy$\pm 1$	Multiclass MCC
Domain experts	0.27	0.73	0.03
Logistic regression	0.35	0.79	0.19
Random forest	0.40	0.83	0.21
CNN	0.50	0.89	0.38
Random guess	0.20 (0.02)	0.55 (0.02)	0.00 (0.02)

Balanced class weights were used to train the classifiers. The bottom row shows the average score (with standard deviation) when random guesses are made.

### Wealth ratings from expert-defined features

Several potentially interesting features were defined based on literature and discussions with experts with extensive knowledge of the Tanzanian context to classify and grade the images for wealth level. The images were rated on three broad criteria: housing features (building types, roofing materials, roofing condition, and building size); landscape features (settlement structure, building density, greenery coverage, dominant land use, and image colour scale); and assets and infrastructure (roads surface quality, roads width, roads coverage, vehicles presence, and farm sizes). Supplementary Table S.2 shows a detailed description of the sub-criteria used for the features. Each image was manually inspected for these features and scored accordingly.

The features, defined in Supplementary Table S.2, were first normalized throughout samples and then used by multinomial logistic regression and random forest learning algorithms. Five-fold cross-validation with randomization was used to estimate the out-of-sample performance. The test outputs from all folds were collected to form confusion matrices for the classifiers in Fig. 3. For logistic regression, the multinomial version was used since it gave the best out-of-sample performance. There are several aspects to what is the appropriate logistic regression model to use here, which are discussed in the supplementary material.

Figure 2c shows the distribution of the wealth levels based on estimates from the logistic regression model using features defined by domain experts. The feature-based logistic regression is much better than the domain experts at estimating the wealth levels, and the MCC value in Table 1 is significantly better than that of random guessing. However, as the confusion matrix (Fig. 2c) shows, the estimation is not great; more than 20% of the estimates are at least two levels away from the correct wealth level. The corresponding number for random forest is 17% .

### CNN model for wealth estimations

The CNN is trained using the night-time-light transfer learning ideas introduced by Jean et al.^8^. Our version of this training is described in detail in Sarmadi et al.^9^. Balanced class weights were use to train the head layer of the CNN. Also here, five-fold stratified cross validation with randomization was used to estimate the out-of-sample performance. The confusion matrix in Fig. 3 shows that the CNN is significantly better at estimating the wealth levels than the domain experts and the feature-based model. The distribution of the wealth levels also matches the ground truth better than any of the other models, as shown in Fig. 2d.

### Comparisons of model predictions

Table 1 compares the wealth estimates from the four approaches using three metrics: *Accuracy*; *Accuracy*$\pm 1$; *Multiclass MCC*. Accuracy measures the fraction of correctly estimated wealth levels. Accuracy$\pm 1$ measures the fraction of correctly estimated wealth levels when an estimate is counted as correct if it is within one wealth level away from the correct one. The multiclass MCC measures the correlation between the correct wealth levels and the estimated wealth levels. The multiclass MCC varies between -1 and +1, with the latter indicating perfect agreement with the ground truth and the former indicating perfect disagreement with the ground truth. Depending on the distribution of the classes, the minimum value of MCC can be larger than -1.

The confusion matrices in Fig. 3 show qualitatively that the CNN is better than the feature-based logistic regression and the random forest, which in turn are better than the domain experts. This is also quantitatively confirmed by the numbers in Table 1. This can be further analyzed by computing the two-class MCC values for the four possible dichotomies. These are shown in Table 2, where 1 denotes *Poorest*, 2 denotes *Poor*, 3 denotes *Middle*, 4 denotes *Rich*, and 5 denotes *Richest*. Table 2 shows that the domain experts are no better than random guessing when it comes to separating the two extreme classes, Poorest and Richest, from the other. This, again, confirms the domain experts’ tendency to go for more average, or central, wealth categories. Neither are the feature-based logistic regression and random forest classifiers much better than random guessing for separating the Poorest from the others, which indicates that the feature set suggested by the domain experts is insufficient for properly recognizing the poorest villages. The CNN-based estimator is significantly better than random guessing for all dichotomies, and also a lot better than both the domain experts and the feature-based Logistic Regression.

**Table 2:** Binary Matthews Correlation Coefficient (MCC) scores for domain experts, logistic regression, random forest, and CNN-based classifiers when dichotomies are done between different wealth classes.

Binary MCC	1 vs. 2-5	1-2 vs. 3-5	1-3 vs. 4-5	1-4 vs. 5
Domain experts	0.05	0.19	0.24	0.03
Logistic regression	0.10	0.29	0.53	0.51
Random forest	0.09	0.22	0.51	0.55
CNN	0.19	0.53	0.68	0.71
Random guess	0.00 (0.04)	0.00 (0.04)	0.00 (0.04)	0.00 (0.04)

Balanced class weights were used to train the classifiers. The bottom row shows the average score (with standard deviation) when random guesses are made.

### Importance of the expert-defined features

**Fig. 4:**
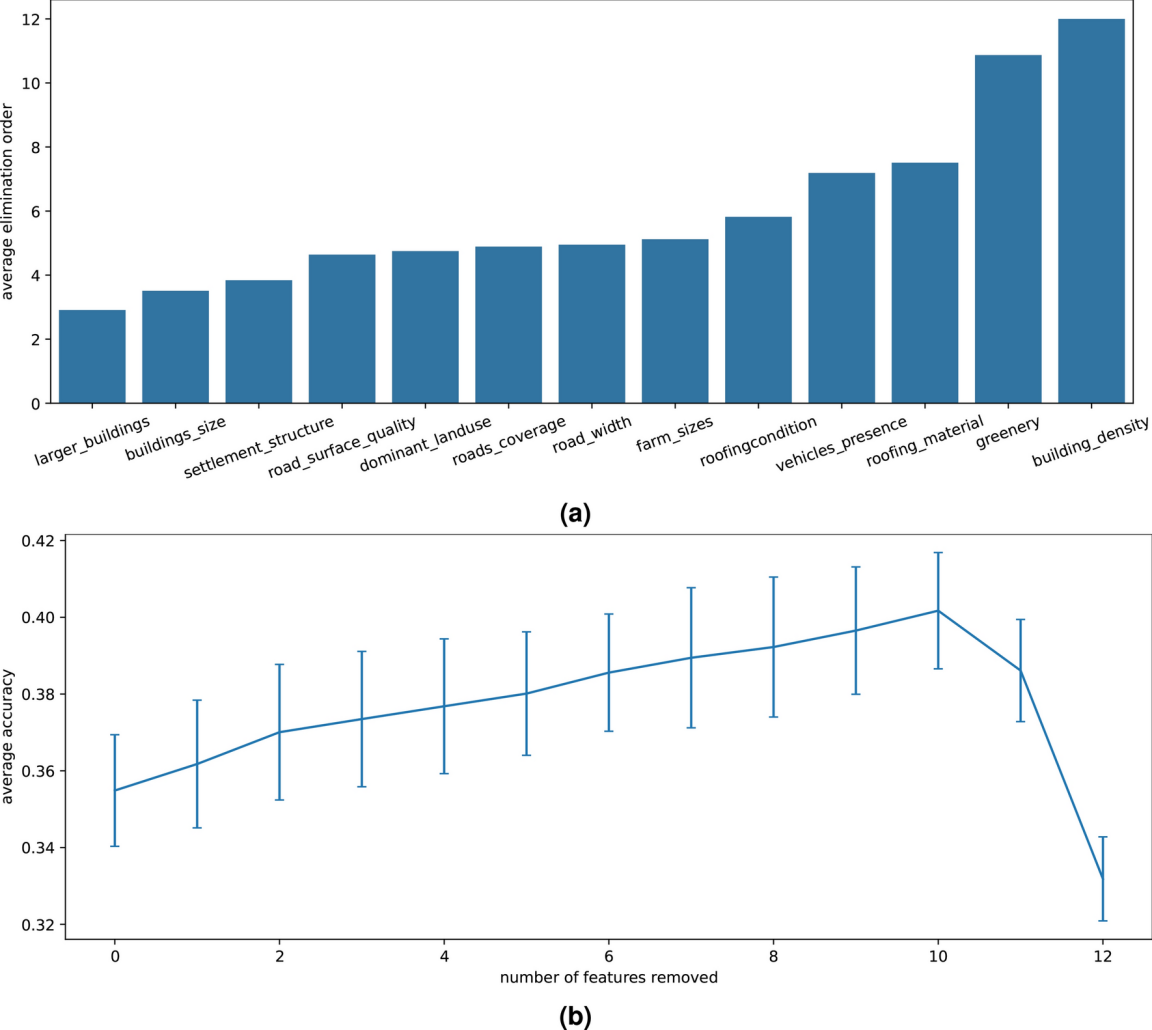
**a** Average removal ranking of the features and **b** average performance during backward elimination when the image_colour feature is not used. Results are for samples with balanced weights across classes and an average of 100 backward elimination experiments. Error bars indicate standard deviation.

We used backward elimination to gauge the importance of the expert-defined features for the multinomial logistic regression. This was done on a reduced feature set; the feature *image color* was removed since it was very similar to the feature *greenery*. Figure 4 shows the result of 100 repeated experiments with 5-fold cross validation and sample randomization among the folds, where the features were removed one-by-one. To make sure the features have equal influence on all wealth classes, we calculated accuracy values with balanced class weights. Interestingly, the accuracy of the wealth prediction initially increases as features are removed.

Figure 4b shows how the accuracy of the prediction increases as features are removed, until only three features are left (10 out of 13 features have been removed) when the accuracy drops sharply. Figure 4a shows how long a feature survives in the backward elimination game: the y-axis shows the average removal round a feature is removed. For example, the feature *building density* always survives to be the last round since it always remains after 12 features have been removed. This means that *building density* is the most important feature in the wealth estimation. The second most important feature is *greenery*, which usually remains after 11 features have been removed. The third most important feature varies between *roofing material*, *vehicles presence*, and *roofing condition*.

In this case, the choice of logistic regression method (multinomial, binomial, or ordinal) influences the feature importance analysis results. This is discussed and illustrated in the Supplementary Material S.5 where the correlations between features (see Fig. S.2), and weight-based and elimination-based feature importance for class dichotomies (see Fig. S.3 and S.4) are explored.

Random forest was also tried along with permutation feature importance calculation to test if it gave different results for the feature importance. These results are shown in Supplementary Material S.5.4 and Table S.3.

### Feature importance for domain expert ratings

The domain experts’ tendency to choose more average wealth levels could possibly be explained by them basing their estimate on a feature that displays more similar values across the sites. To test for this, we explored how the expert-defined features related to the domain expert ratings. For this purpose, an ordinal logistic regression predictor was trained using the maximum likelihood algorithm. The samples were re-weighted according to the survey class weights for consistency with our other feature importance experiments, and a z-test was employed to determine the two sided *p*-values for each feature. The results are shown in Table 3; the four features with *p*-values below 0.05 are highlighted in the table. Comparing this to Fig. 4 and considering the fact that the order of the features varies with different runs (only *building density* and *greenery* are stable at the top) shows that the domain experts and the logistic regression consider very similar features in the images. Consequently, different feature sets do not explain the difference between the domain expert and the feature based logistic regression estimates of wealth. A human tendency to choose more average estimates is more likely.

**Table 3:** Coefficients, standard deviation (SD) of the error, z-values, and *p*-values for different features calculated using an ordinal logistic regression classifier.

Feature	Coefficient	SD	z	*p*-value
Building size	-0.0158	0.074	-0.213	0.831
Roofing material	0.1189	0.085	1.404	0.160
**Roofing condition**	**-0.2053**	**0.073**	**-2.800**	**0.005**
Taller buildings	-0.1153	0.082	-1.399	0.162
Settlement structure	-0.0057	0.079	-0.072	0.942
**Building density**	**0.6994**	**0.096**	**7.273**	**0.000**
**Greenery**	**0.4609**	**0.082**	**5.590**	**0.000**
Dominant land use	-0.1025	0.081	-1.266	0.205
Road surface quality	0.0101	0.085	0.119	0.905
Road width	0.0622	0.066	0.938	0.348
**Road coverage**	**0.4347**	**0.083**	**5.263**	**0.000**
Vehicles presence	0.1609	0.095	1.694	0.090
Farm sizes	-0.0202	0.078	-0.258	0.796

The features with *p*-values below 0.05 are highlighted.

## Discussion

Human bias in ranking and a tendency to go for central, or average, options has been observed in other fields. A short review of the research on this “central tendency bias”, dating back to 1910, can be found in Akbari et al.^10^. Interestingly, Crosetto et al.^11^ show that this bias persists even if subjects are informed that options should be uniformly distributed. Thus, it is not surprising to see it in wealth estimation from images as well. However, in this paper we compare the results from domain experts to that of the machine learning-based methods which has been a common approach in, for example, medicine and AI^12^. This was done on a spectrum where at one end there is total expert opinion and at the other end the total decision of machine learning approach based on satellite images. While in the middle there is a machine learning method that uses features defined and evaluated by domain experts. The results showed a clear and separable difference in performance that is worst on the human end and best at the machine learning end of the spectrum. This indicates that not only can domain experts not be fully trusted at ranking but even their definition and evaluation of features results in sub-par wealth estimation. One could extend this to other areas where a decision needs to be made by a human domain expert on satellite images. This, of course, does not mean that there is no bias in methods based on machine learning, but this bias originates with the data, which can be handled. The human bias of domain experts is more complicated to compensate for.

The results also show that the potential to leverage deep machine learning and satellite imagery to gain new insights in the domain of poverty and welfare is significant. This potential is closely linked to recent debates on explainability within the field of computer science, where there is a high demand for understanding the operational mechanisms and decision-making processes of specific models^13^. Explainability is essential to ensure the scientific value of these models, thereby deepening our understanding and facilitating new scientific discoveries. Our results demonstrate that ML methods can identify features that result in much better estimations than those suggested by domain experts. This underscores the capability of machine learning to uncover new knowledge, which can aid human experts in their understanding of poverty and welfare dynamics. This highlights a crucial synergy between advanced ML techniques and human expertise, where AI not only enhances existing knowledge but also reveals previously unrecognized patterns and insights from satellite imagery data, paving the way for new discoveries in the field.

A different aspect from which we could look at this problem is explainability. Features that are defined by humans give more potential to understand how the machine learning tool is perform in the tasks. However, our experiments show that those manually-defined features might not interact constructively and they could have a significant overlap between them. This means that even for explainable features there is a need of machine learning based verification that identifies how helpful they are and which group of features could perform well when selected together. Notwithstanding this argument, there are ad-hoc explainability methods that could be used with non-explainable methods such as CNN which could give insights into the working of the model, even though they are not very straightforward to deduce knowledge from. We suggest that the most interesting finding of our paper is how good the CNN classifier works. This is because the CNN based network takes images that are much lower resolution than those seen by the domain experts. The domain experts had even the freedom to zoom in and out on the satellite imagery while the CNN had a fixed resolution. This suggests that there are a set of features on a higher scale which are not known or properly measurable by the experts but the CNN feature extractor has found and engineered them. This is a very interesting fact that could motivate further development of explainability methods in conjunction with CNN networks to give us more insight into useful features that could indicate poverty.

The emergence of building density, and roofing materials and conditions as key markers of higher wealth quintiles is both unsurprising and encouraging. These features align with the expected indicators of wealth, reflecting improved living standards and investment in durable housing. Conversely, other anticipated features, such as building sizes, road surface quality, coverage, width, and settlement structure, turned out to be less significant in this context. While these attributes are often highlighted in discussions of slum characteristics, implying lower wealth, our findings suggest that their importance may vary across different settings.

These results are consistent with field-based observations, such as those in Östberg’s longitudinal study in Tanzania, which traced livelihood changes and poverty dynamics over 25 years^14^. The study found that substantial wealth gains in two Tanzanian villages were driven by local agricultural practices, particularly sunflower farming as a cash crop. Despite previous perceptions of small-scale farming in Tanzania as stagnant, the area of land farmed per family nearly doubled, leading to increased local wealth. Investments in mechanized farming, housing improvements, education, livestock, and consumer goods were notable. Improved infrastructure and local entrepreneurship played significant roles in transforming these communities.

Östberg et al.’s study underscores the value of examining seemingly mundane features-such as roofing materials and farm sizes-to understand wealth transitions. The observed shift from reliance on casual labor to self-sufficiency through farming marks a significant socio-economic change. However, this progress came at an environmental cost, with deforestation replacing village forests with farms, raising concerns about climate change impacts. Our findings, showing the relative importance of different structural features, provide new insights into the complex interplay between physical infrastructure and socio-economic status, offering valuable perspectives for future poverty alleviation efforts.

The results of our study demonstrate that convolutional neural networks (CNNs) are capable of surpassing human experts in the analysis of satellite images by identifying more effective features for decision-making. Importantly, the performance of CNNs suggests their potential not only in automating tasks but also in contributing to scientific discovery by revealing unknown or overlooked patterns in data. A recent review shows that the research field of poverty estimation from satellite images needs much more research on explaining the CNN decisions^15^.

CNNs operate by extracting and processing layers of features from raw data, often discovering intricate structures that are not immediately apparent to human observers. These features, while highly effective in specific tasks such as wealth estimation from satellite images, also carry broader implications. For instance, CNNs might identify subtle variations in landscape patterns that correlate with economic indicators, features that human experts have not previously recognized. By studying these machine-identified features, researchers can hypothesize new causal relationships or refine existing theories about socio-economic determinants.

The superior ability of CNNs to discern and leverage such features can transform how domain experts approach their fields. Researchers can analyze the features that CNNs prioritize to understand why these are more predictive and what this suggests about the underlying phenomena. This process can lead to a revision of current models or theories in the field, facilitating a deeper understanding of key variables and their interactions. Additionally, these insights can guide the development of new experimental designs or data collection strategies, aiming to further test and validate the findings initiated by machine learning analyses^13^.

To harness these insights effectively, an integrative approach can be adopted where the outputs of CNNs are not just used for task performance but also studied for their scientific implications. By integrating machine learning findings with traditional research methods, domain experts can explore new hypotheses and expand the boundaries of domain knowledge. For example, in environmental science, features identified by CNNs from satellite imagery could lead to new insights into climate change impacts or biodiversity assessments.

Future research should focus on mechanisms to decode and interpret the feature selection processes of CNNs, making these more accessible and understandable to human experts. This could involve the development of visualization tools that map how CNNs process data and identify features, or the creation of interfaces that allow experts to interact with and query the model about its decisions. Such tools will help bridge the gap between machine learning outputs and human cognitive processes, fostering a productive dialogue between them.

## Data and methods

### The Tanzania 2015 DHS dataset

The ground truth data is derived from the 2015/2016 Tanzania Demographic and Health Survey (TDHS) dataset, which is the sixth in the DHS series for the country. While the overall DHS programme is concerned with collecting and monitoring data on population, health and nutrition, it also collects data on households’ living standards. This paper uses data on the welfare status of households surveyed in this campaign. The $6^{th}$ TDHS uses a nationally-representative sample of 12,563 households, grouped into 608 clusters across the 30 regions of the country. For the present paper, the unit of analysis is the *cluster*, which ranges in sample size from 12 to 22, with a mean of 21 (SD=1.55), households per cluster. More details about the dataset and its usage are provided in the Supplementary Material, Section S.1.

### Aerial image extraction and online survey

The Google Maps Platform hosts a set of APIs through which developers can retrieve data from. Examples of the used images are shown in the Supplementary Material Fig. S.1. The site coordinates were fed into an R-script accessing the Google Maps platform to download corresponding ultra-high-resolution images for clusters. In total, 608 images were downloaded at zoom level 18. This zoom level corresponds to a pixel size of about 0.6 meters. Details regarding the survey of domain experts on aerial images are provided in the Supplementary Material, Section S.2.

### Classifiers on expert-defined features

Multinomial logistic regression was trained with the Limited Memory Broyden-Fletcher-Goldfarb-Shanno algorithm^16^. At each iteration of the 5-fold stratified cross-validation, the training slice was used with another 5-fold cross-validation algorithm to find the optimal $l_2$ regularisation coefficient, in logarithmic steps between $10^{-4}$ and $10^4$.

Each random forest classifier was an ensemble of 100 decisions trees. The Gini impurity criterion was employed to split the nodes and the number of features randomly chosen to split them was set to the square root of the feature set’s cardinality. No maximum depth was set for the trees hence the branches grew to decrease impurity with at least two samples on the split nodes.

### Data and models used for CNN predictions

Convolutional neural network (CNN) models are the current state of the art for estimating wealth levels from daytime satellite images. A detailed description of the model building is given in^9^. The CNN backbone is trained via transfer learning on the MobileNetV2^17^ model, which is pretrained on ImageNet data. The transfer learning means that the MobileNetV2 CNN model is fine-tuned to estimate the $\log _{10}(1+x)$ of the night time light intensity (*x*) from the corresponding daylight satellite images measured in nW/$\hbox {cm}^2$/sr. After this, the CNN is able to estimate the night-time light values out-of-sample with an $R^2$ score of 0.81 for the 608 clusters used in our study. After the transfer learning, the dense head layer of the CNN is retrained as a ridge classification layer with five outputs, corresponding to the five wealth categories. When predicting, the output with the maximum value is selected as the wealth label. This task is done in the framework of the TensorFlowV2 library (www.tensorflow.org). There are two differences compared to the network described in^9^. First, we use a slightly updated image set for the training where we allow a maximum of only 25% of the image to be cloudy. The second difference is that the dense head layer of the network is trained as a classification layer.

We used the $3\times 3$ variation of the model in^9^, meaning that we use the fully convolutional CNN and give the $3\times 3$ neighborhood of the corresponding image as input to extract features. It is important to note that the Sentinel-2 daytime satellite images used by our CNN are much lower resolution than the high-resolution images shown to the domain experts, and also lower resolution than the satellite images used by Jean et al.^8^. Still, the wealth estimation accuracy is on par with the results reported in Jean et al.^8^. The Sentinel-2 dataset has a pixel length corresponding to 10 meters. The ridge regression based classification algorithm used a 5-fold cross validation within each training slice of the outer 5-fold cross validation splits to get the regularisation coefficient for each class.

## Supplementary Information

Supplementary Information.

## Data Availability

DHS Survey data for Tanzania 2015 can be found at: https://www.dhsprogram.com/data/dataset/Tanzania_Standard-DHS_2015.cfm. Sentinel-2 daylight and night light images for CNN transfer-learning were downloaded from Google Earth Engine data catalog: https://developers.google.com/earth-engine/datasets. Sentinel 2 daylight images were extracted from earth engine image collection COPERNICUS/S2 and night light intensities were extracted from earth engine image collection NOAA/VIIRS/DNB/MONTHLY_V1/VCMCFG. The rest of the data is available from the corresponding author on reasonable request.
