# Supplementary material for: Human bias and CNNs’ superior insights in satellite based poverty mapping
Source: Sci Rep. 2024 Oct 2;14:22878. doi: 10.1038/s41598-024-74150-9 (PMC11447245; doi:10.1038/s41598-024-74150-9)
Supplement: Supplementary file 1 — Supplementary Information. [file 41598_2024_74150_MOESM1_ESM.pdf]

Human Bias and CNNs' Superior Insights in Satellite Based Poverty Mapping

Hamid Sarmadi[2,*], Ibrahim Wahab[1], Ola Hall[1], Thorsteinn Rögnvaldsson[2], and Mattias Ohlsson[2,3]

[*]hamid.sarmadi@hh.se
[1]Department of Human Geography, Lund University, Sweden
[2]Centre for Applied Intelligent Systems Research (CAISR), Halmstad University, Sweden
[3]Centre for Environmental and Climate Science, Lund University, Sweden

S Supplementary Material

S.1 Tanzania 2015 DHS dataset

S.1.1 Indicator variables and the wealth index

The DHS Wealth Index is an asset-based index designed to compare relative, rather than absolute, economic wellbeing of households independently from education and health. The index is based on households' ownership of assets (such as radio, televisions, telephones, refrigerators, computers, bicycles, motorcycles, cars, bank accounts, house and lands); access to services (such as electricity, toilet facilities, and water sources, and sources of energy for cooking); and building materials (primarily flooring, walls and roofing materials). Responses to questions on assets, services, and enumerators' observations of building construction materials in the DHS survey are considered more reliable than self-reported income- and expenditure-based estimation of welfare and economic wellbeing [7]. The combined wealth index of clusters, the dependent variable, has been grouped into quintiles: *poorer*, *poor*, *average*, *wealthy* and *wealthier*. While others, such as [6] and [3], who have used this dataset have regrouped these quintiles into poor and non-poor, we opted to maintain the original categorizations to bring further nuance to the relative poverty ratings. At each cluster we calculate the mean of the combined wealth index and see which of the quintiles it belongs to. Consequently, since the quintiles are calculated for households and not clusters the number of clusters that fall in different quintiles is not necessarily the same.

S.1.2 Geolocation of clusters

While it is now standard practice for DHS datasets to come with location data of clusters, the DHS programme anonymises it by adding noise to the location variable for ethical reasons. In the current dataset, urban clusters are displaced by a distance of up to two kilometres and up to five kilometres for rural clusters, with a further randomly selected one per cent of the rural clusters displaced by up to ten kilometres [2]. This introduces uncertainties in the predictions as we matched coordinates with clusters. We overcome this by "correcting" the GPS data to

the most likely location in an iterative manner on Google Maps. We achieve this by ensuring that we follow the guidelines for adding noise to the locations. That is, for rural locations, we assume that the nearest settlement within 5 km is the most likely "actual" cluster and expand the search area to 10 km in the few cases where we could not find any possible settlement in the 5km radius. We then use the "corrected coordinates" of clusters to extract cluster images.

S.2 Satellite images and online survey

The Google Maps Platform hosts a set of APIs through which developers can retrieve data from the platform. It is notoriously difficult to find metadata about images provided in the service. Generally, images are from different sensors and combined into a mosaic of images taken over multiple periods, with seams between different images being possible to identify sometimes. You can see some examples of the used images in Fig. S.1. The corrected coordinates were fed into an R-script accessing the Google Maps platform and downloading corresponding ultra-high-resolution images. In total, 608 images were downloaded at zoom level 18. That corresponds to a pixel size of about 0.6 meters.

The extracted images were then fed into the web-based survey platform (`https://predict.gis.lu.se/`). A pilot for the survey ran for two weeks using a group of Master's students at Lund University to test the resilience of the platform as well as the validity and reliability of our experimental design before the main survey went live between March 1 and May 31, 2022. Our approach was to send personalized invitations to experts within our networks who have fieldwork experience in Africa in the area of development research, broadly defined. We had contemplated sending a general invitation to our networks or asking respondents to forward invitations to other experts they deem qualified but eventually decided against these two options. First, we supposed that a general, unsolicited email might not garner the same response rate as a personal one. Secondly, we wanted to control the sample of human experts who rated the images. Each expert is first presented with a batch of 30 images in a sequential manner and asked to rate each one as either *poorer*, *poor*, *average*, *wealthy* or *wealthier*, to be comparable with the DHS quintiles. Raters were allowed to rate additional batches of 30 images if they so wished. Overall, the web portal received 2,174 ratings from 102 experts. The median rating for each cluster image was also taken as representing the relative poverty score for the cluster.

S.3 Domain Experts

As Table S.1 shows, the sampled domain experts come with significant experience. This shows in not only their educational attainment and age distribution – 43% are 45 or more years old and 50% hold a Ph.D. – but even more importantly, that half of the respondents (N=51) have more than 10 years of fieldwork experience in SSA (South Saharan Africa). In terms of the region of experience, 48% have Tanzania, Eastern, or Southern Africa as their primary area of fieldwork experience.

Of the 608 clusters for the 2015 TDHS dataset, 428 were classified as rural with the remaining 180 classified as urban. By our iterative approach to geolocate the clusters, we estimate average dislocation of 3.09 (SD=2.66) km for rural areas and 1.15 (SD=0.77) for urban clusters. These fall within the DHS geographical displacement guidelines. Fig. 2 (b) and (a) show the distribution of the wealth quintiles based on ratings by our domain experts and the DHS dataset, respectively. While comparable in terms of the quintile categorisation, it must be noted that the unit of analysis for the DHS survey is the household (N=12,563) which then had to be aggregated to the cluster level to match the web-based survey where the unit of analysis is the cluster level (N=608).

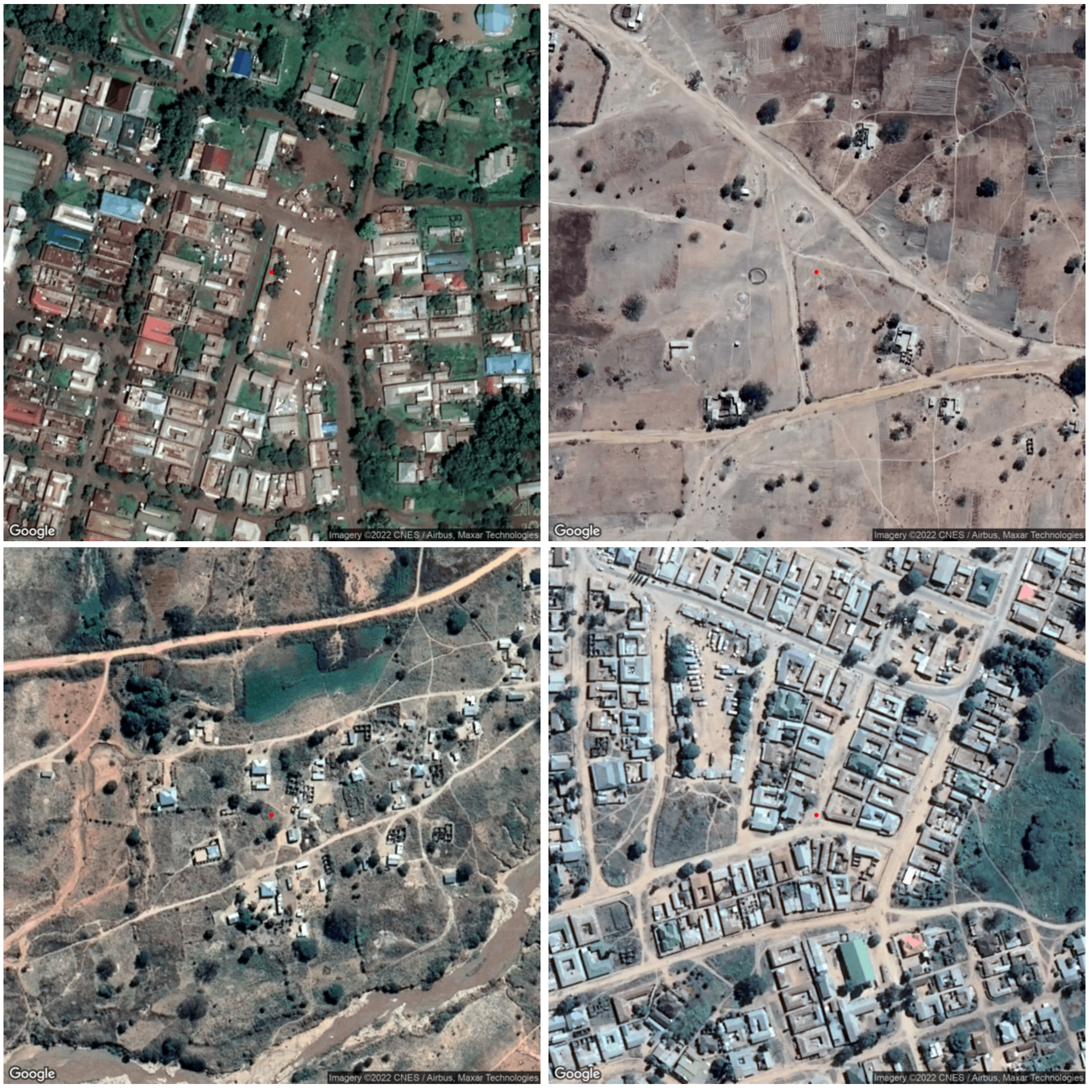

Figure S.1: Samples of images from four different locations in Tanzania. These images were downloaded from Google Maps Static API (`https://developers.google.com/maps/documentation/maps-static`). Note that images are reduced by 40% from original size.

Table S.1: Summary statistics of web-based survey respondents (a total of 102 respondents)

Variable	Categories	Distribution
Age of respondents	Up to 35 yrs old	31
	36 - 55 yrs old	53
	56 yrs old and above	18
Gender	Females	23
	Males	77
	Non-binary	2
Educational qualifications	Bachelors	17
	Masters	34
	Ph.D.	51
Length of experience (years)	1 - 5 yrs	24
	6 - 10 yrs	27
	11 - 20 yrs	31
	Above 20 yrs	20
Region of experience	Tanzania	14
	Central and West Africa	53
	Southern and Eastern Africa	35

S.4 Expert Defined features

To shed light on the correlates of the ratings by experts, a criterion was developed, based on literature and discussions with experts with extensive knowledge of the Tanzanian context to classify and grade the images based on pertinent spatial features which could be discerned. On this, we rated the images on three broad criteria, namely, housing features (building types, roofing materials, roofing condition, and buildings' size); landscape features (settlement structure, building density, greenery coverage, dominant land use, and image colour scale) and assets and infrastructure (roads surface quality, roads width, roads coverage, vehicles presence, and farm sizes. Table S.2 presents a detailed description of the sub-criteria used for the classification. Each image was manually inspected for these qualities and scored accordingly to find the correlates of the ratings by the domain experts.

Table S.2: Literature and expert-based correlate features in images.

Broad criteria	Spatial features	Categories	Codes
HOUSING FEATURES	Size of buildings	single units	1
		multiple units	2
		unclassified	99
	Roofing Material	thatch roofing	1
		aluminium roofing	2
		slate roofing	3
		unclassified	99
	Roofing Condition	uncompleted	1
		old	2

		new	3
	Presence of tall buildings	no	1
		yes	2
		unclassified	99
LANDSCAPE FEATURES	Structure of settlements	clustered	1
		scattered	2
		gridded	3
	Density of built environment	low coverage (less than 25%)	1
		medium coverage (between 25–74%)	2
		high coverage (greater than 75%)	3
	Presence of trees/greenery	low (below 25%)	1
		medium (25–75%)	2
		high (above 75%)	3
	Dominant land use	bare land	1
		agricultural	2
		built up	3
		industrial	4
		commercial	5
		unclassified	99
	Image colour	brownish	1
		yellowish	2
		greenish	3
ASSETS AND INFRASTRUCTURE	Presence of vehicles	no vehicles visible	0
		few vehicles (1–2 visible)	1
		many vehicles (more than 2 visible)	2
	Road surface quality	no roads	0
		untarred roads	1
		tarred roads	2
	Road width	small	1
		medium	2
		large	3
		unclassified	99
	Farm sizes	small farms	1
		large farms	2
		no farms	3
	Road coverage	low	1
		medium	2
		high	3

All three datasets – the DHS survey data, experts' rating of relative poverty, and researchers' scoring of discernible features in images – were merged into one dataset using the cluster code as the link.

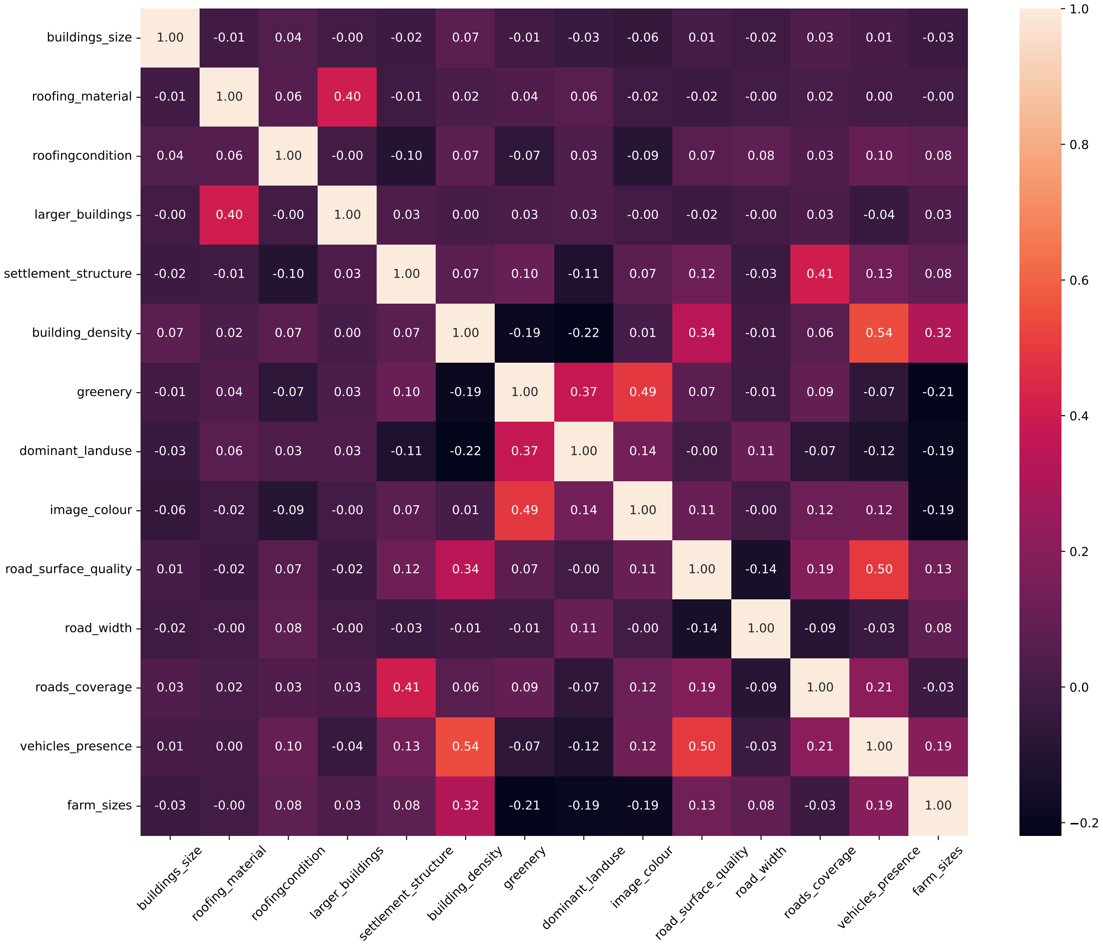

Figure S.2: Correlations between manually defined features extracted for all the test clusters.

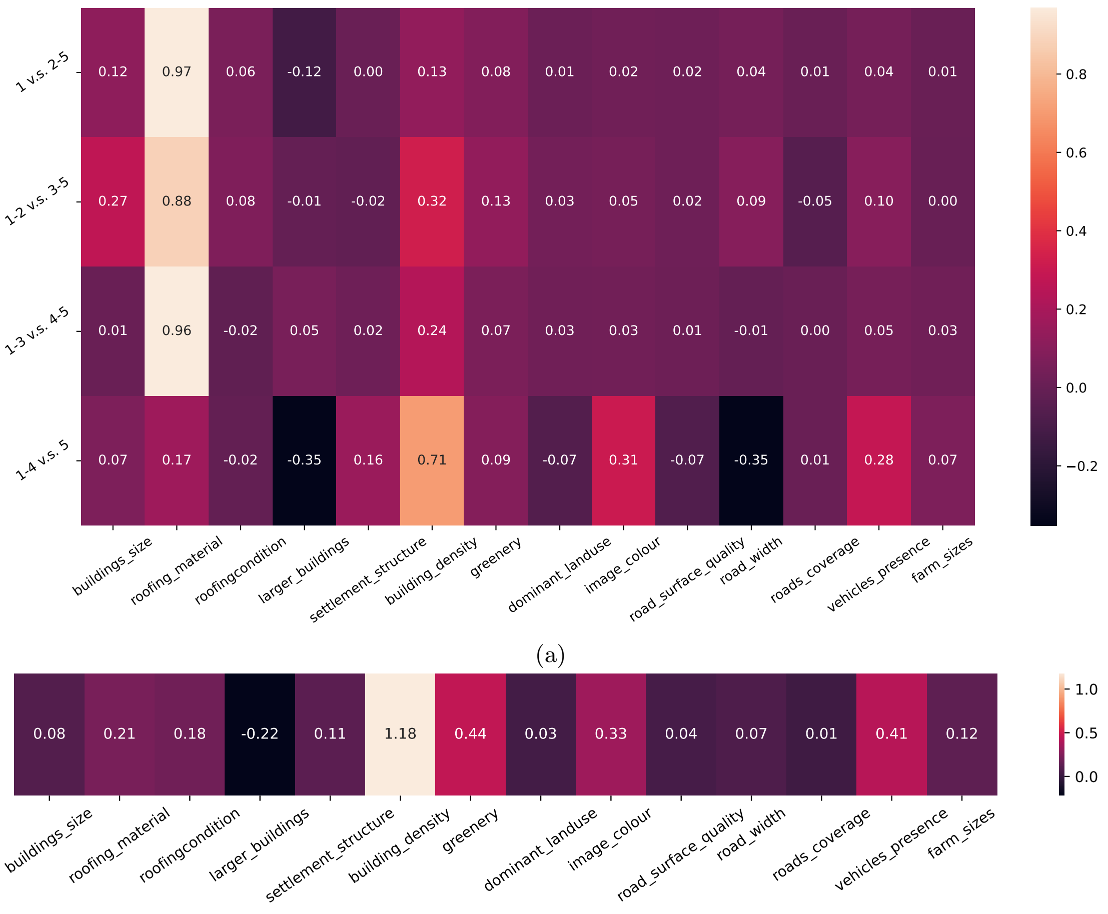

(a)

(b)

Figure S.3: Average weight for each feature in the (a) multinomial and (b) ordinal logistic regression classifiers accross 100 experiments.

S.5 Feature Correlation and Importance

S.5.1 Correlation between feature values

Figure S.2 shows how related the expert defined features are to each other in terms of Pearson correlation coefficients, calculated using the 608 clusters in our Tanzania dataset. The highest correlation is between *building density* and *vehicle presence*, and *building density* and *road surface quality*. After that, the highest correlation is between *image colour* and *greenery*. In the case of *image colour* and *greenery* the causal dependence is obvious hence we suggest that *image colour* is an auxiliary feature.

S.5.2 Logistic regression weights

The logistic regression model is linear, which means that the weights (the parameters) of the model reflect the importance of the features. Because the five wealth categories are imbalanced regarding the number of samples we applied class weights to equalize them for training and

also classification accuracy.

Here, care must be taken as to what logistic regression method to use. A multinomial logistic regression separates each class from the others and thus produces the feature weights that are most unique for each particular class (in this case, five sets of weights). This may not reflect what is important overall for wealth and poverty. On the other hand, an ordinal logistic regression [4] will yield only one set of weights, considering that the classes are ordinal. This will be an overall set of weights, which also reflects the wealth and poverty relation better than the multinomial logistic regression. For this experiment, we opted for a choice in-between the multinomial and ordinal, which both gave weights that reflect wealth and poverty and more detailed information. This was achieved by doing binomial logistic regression for each of the four dichotomies described earlier (poorest vs. the others, poorest and poor vs the others, etc.).

For each dichotomy, a total of 100 logistic regression experiments were done, with variations in the data samples, and the feature weights were averaged over the experiments. All features were normalized to have the same scale. The average weights are presented in Fig. S.3a, which shows that *roofing material* is the most influential feature for all dichotomies except separating the richest sites from the others (1-4 vs. 5), where *building density* is the most influential feature. A general observation is that the extreme dichotomies (1 vs. 2-5 and 1-4 vs. 5) are different from the more central dichotomies. The feature *roofing material* is more prominent in the separation of the poorest from the others. These results can be compared to those achieved with an ordinal logistic regression classifier, in Fig. S.3b where *building density* appears to be overall most influential.

S.5.3 Backward elimination of features

To further analyze the features' relative importance we used backward elimination for the logistic regression classifier on the dichotomies. The process involves removing the worst performing feature, one at a time, to see which features remain at later stages. The *image colour* feature was removed since it gave very similar results to *greenery.* The results are presented in Fig. S.4, where Fig. S.4a shows the average elimination order, and Fig. S.4b shows the average classification accuracy achieved with that number of features. A feature that has a value of 12 for average elimination order survives to the end in all backward elimination experiments, a feature that has a value of 11 is usually the second last to be removed, and so on.

The *building density* feature is the most important feature across the board, which agrees with the ordinal logistic regression weights in Fig. S.3b. The feature *greenery* is second most important for separating the poor classes from the others, followed by *roofing material* and *roofing condition.* For the richest, the feature *vehicles presence* appears very important, together with *greenery.*

The accuracy plots in Fig. S.4b show that it is often enough with 2-3 features to get good accuracy. It appears that the separation of rich and richest from the other sites requires most features (3), whereas the extreme dichotomies (poorest vs. others, and richest vs. others) get good accuracy with only two features.

S.5.4 Feature importance measured with random forest

A random forest predictor was also constructed for the domain expert features. A feature importance analysis based on the permutation importance [1] was done on this random forest predictor. For consistency, the image colour feature was dropped and balanced samples were used for training and evaluation. 100 different experiments with different random sampling were performed. In each experiment 5-fold cross validation was employed and within every fold, 10 permutation experiments were executed and results were averaged over experiments,

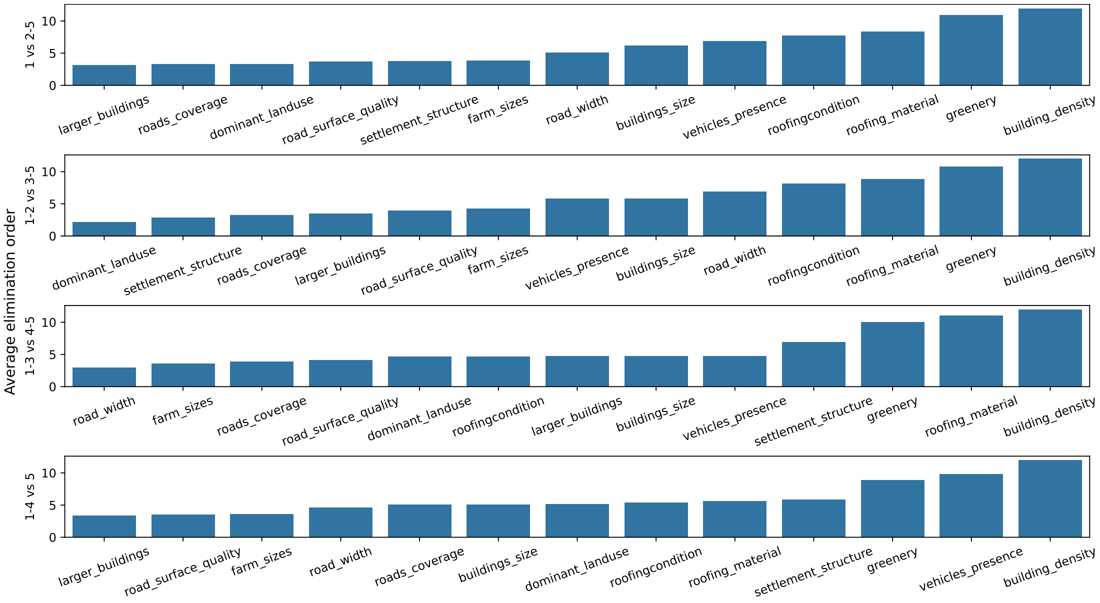

(a)

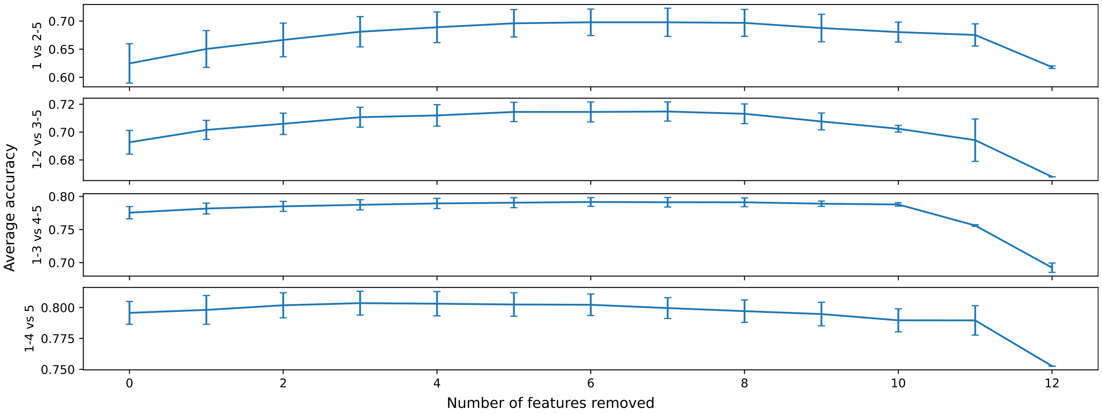

(b)

Figure S.4: (a) Average elimination feature ranking and (b) change in classification accuracy from the backward elimination procedure done on the dichotomy classifiers. The results are the average of 100 separate experiments with 5-fold cross-validation.

Feature	Mean (%)	SD (%)	Mean/SD
Building size	-0.66	1.88	-0.35
**Roofing material**	**3.40**	**1.44**	**2.36**
**Roofing condition**	**1.05**	**2.34**	**0.45**
Taller buildings	-1.45	1.81	-0.80
Settlement structure	-1.15	2.14	-0.54
**Building density**	**7.03**	**2.52**	**2.79**
**Greenery**	**1.45**	**2.12**	**0.68**
Dominant land use	0.27	2.21	0.12
Road surface quality	0.21	1.76	0.12
Road width	-0.63	1.62	-0.39
Road coverage	-0.27	1.69	-0.16
**Vehicles presence**	**2.34**	**2.31**	**1.01**
Farm sizes	-0.61	2.05	-0.30

Table S.3: Mean and standard deviation (SD) of decrease in accuracy of the random forest classifier in the feature permutation experiments. The results are the averages of 5-fold of cross validation. The top five are boldfaced. (A negative mean value means that the accuracy increased when the feature was removed.)

fold, and permutation experiments. Table S.3 shows that the top five features with the highest mean reduction in accuracy in the random forest experiment are the same as the top five features estimated using backward elimination and logistic regression (Fig. 4 in the main paper). *Building density* comes out as clearly most important in both the random forest and the logistic regression experiments, which also agrees with what appears most important for the CNN [5]. The features' order of importance is similar to that of multinomial logistic regression except for the *Greenery* feature, which is second to last in top 5 as opposed to second to first. The logistic regression results on the four dichotomies (Fig. S.4) show that *Greenery* is more important for the poorest categories and less for the wealthy, which could mean that the random forest focuses on wealthier classes. The random forest also has more problems with predicting the poorest category than the logistic regression.

References

[1] André Altmann, Laura Toloşi, Oliver Sander, and Thomas Lengauer. Permutation importance: a corrected feature importance measure. *Bioinformatics*, 26(10):1340–1347, May 2010.

[2] Clara R. Burgert and Debra Prosnitz. Linking DHS household and SPA facility surveys: Data considerations and geospatial methods. September 2014. Number: DHS Spatial Analysis Reports No. 10.

[3] Qing Li, Shuai Yu, Damien Échevin, and Min Fan. Is poverty predictable with machine learning? A study of DHS data from Kyrgyzstan. *Socio-Economic Planning Sciences*, 81:101195, June 2022.

[4] Peter McCullagh. Regression Models for Ordinal Data. *Journal of the Royal Statistical Society. Series B (Methodological)*, 42(2):109–142, 1980. Publisher: [Royal Statistical Society, Wiley].

[5] Hamid Sarmadi, Thorsteinn Rögnvaldsson, Nils Roger Carlsson, Mattias Ohlsson, Ibrahim Wahab, and Ola Hall. Towards Explaining Satellite Based Poverty Predictions with Convolutional Neural Networks. In *2023 IEEE 10th International Conference on Data Science and Advanced Analytics (DSAA)*, pages 1–10, October 2023.

[6] Badiea Shaukat, Sajid Amin Javed, and Waqas Imran. Wealth Index as Substitute to Income and Consumption: Assessment of Household Poverty Determinants Using Demographic and Health Survey Data. *Journal of Poverty*, 24(1):24–44, January 2020. Publisher: Routledge _eprint: https://doi.org/10.1080/10875549.2019.1678550.

[7] Sarah Staveteig and Lindsay Mallick. Intertemporal comparisons of poverty and wealth with DHS data: A harmonized asset index approach. September 2014. Number: DHS Methodological Reports No. 15.
